# Coyotes Hunt Harbor Seal Pups on the California Coast

**DOI:** 10.1002/ecy.70031

**Published:** 2025-02-12

**Authors:** Francis D. Gerraty, Sarah Grimes, Sue Pemberton, Sarah G. Allen, Sarah A. Codde

**Affiliations:** ^1^ Department of Ecology and Evolutionary Biology University of California—Santa Cruz Santa Cruz California USA; ^2^ Noyo Center for Marine Science Fort Bragg California USA; ^3^ California Academy of Sciences San Francisco California USA; ^4^ Point Reyes National Seashore Point Reyes Station California USA

**Keywords:** *Canis latrans*, land‐sea connectivity, marine subsidies, *Phoca vitulina*, pinnipeds, predation

Terrestrial carnivores hunt marine mammals along many coastlines around the world, yet the dynamics of these predator–prey relationships are rarely well characterized (Griffin et al., 2023; Stander, 2019; Way & Horton, 2004). Among marine mammals, pinnipeds (i.e., seals and sea lions) may be particularly vulnerable to land‐based predators when they aggregate onshore to rest, give birth, nurse, breed, molt, avoid marine predators, and thermoregulate (Catenazzi & Donnelly, 2008; Nordstrom, 2002). Many pinniped species exhibit strong site fidelity, so rookery beaches where birthing and breeding take place can generate spatially and temporally predictable prey aggregations for terrestrial predators. These aggregations of large and calorically rich prey can serve as a substantial food source that may lead to shifts in predator behavior, abundance, and species interactions (Roffler et al., 2023; Skinner et al., 1995).

During routine harbor seal (*Phoca vitulina*) population monitoring at a small mainland rookery site at MacKerricher State Beach (Mendocino County, California) during the March–June pupping seasons in 2016 and 2017, we noticed a surprising trend in harbor seal pup mortality. In each year, three harbor seal pups were found in dune vegetation above the high tide line adjacent to the rookery, showing numerous signs of predation‐driven mortality, including marks in the sand indicative of a struggle, drag marks from the lower intertidal to the dune area, massive hemorrhaging in the head and neck region, and skull punctures (Figure 1). This characteristic pattern of pup mortality increased in frequency in 2018 and 2019, when 11 and 12 pups were respectively found showing similar signs of predation. In most cases, pup carcasses were found within an approximately 25‐m^2^ area of dune vegetation, with skulls disarticulated from the rest of the body at the C1 vertebra, and skulls were missing on several occasions. This unusual pup mortality pattern has been documented at the MacKerricher rookery site every year since 2016—including four cases in 2024—for a total of 55 suspected predation events (Figure 2; Appendix S1: Table S1).

**FIGURE 1:**
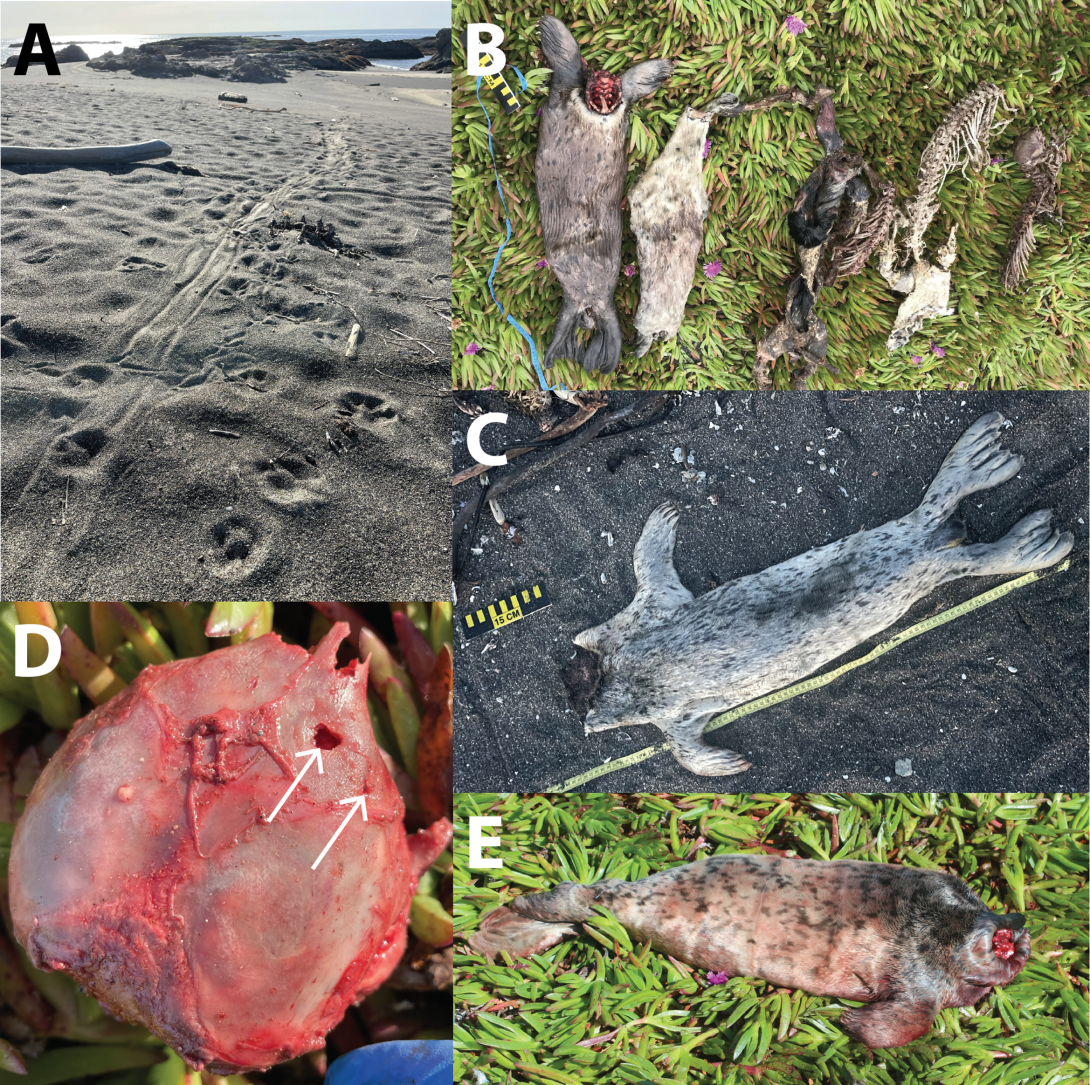
Regularly documented evidence of coyotes hunting harbor seal pups at MacKerricher State Beach includes (A) coyote tracks and drag marks indicating coyote ambush predation and seal pup carcass removal from rookery; (B, C, E) skull disarticulation and, in some cases, skull removal from the carcass deposition site; (B) consistent deposition of seal carcasses within a small patch of dune vegetation; and (D) skull punctures consistent with coyote predation. Photo credit: (A, B, C) Sarah Grimes, (D, E) Francis D. Gerraty.

**FIGURE 2:**
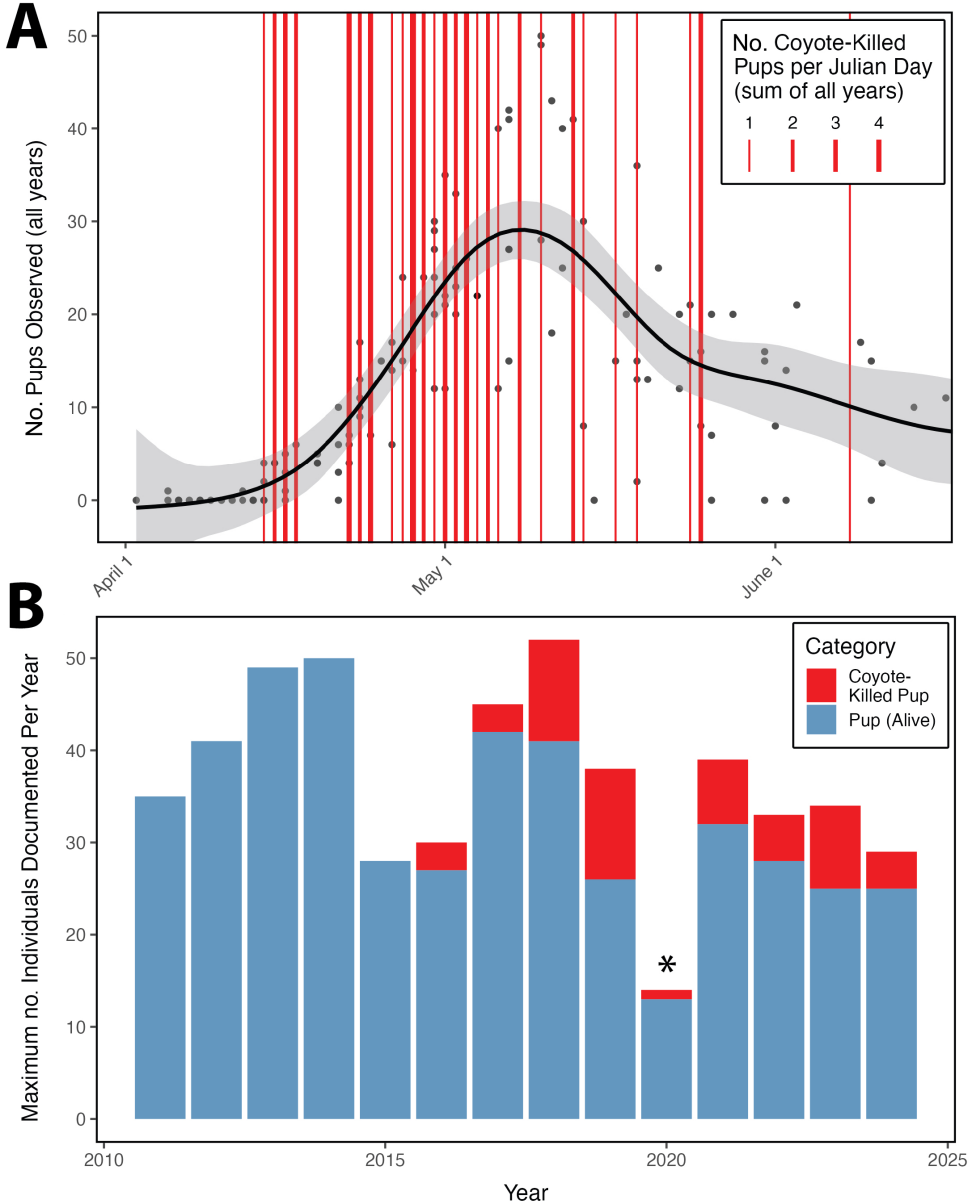
(A) Seasonality of harbor seal pupping and coyote predation at MacKerricher State Beach. The black points represent the total number of pups counted (pooled across 2011–2018), the black line is a generalized additive model fitted to the pup counts, and the red vertical lines represent suspected and confirmed coyote predation events on harbor seal pups. (B) Abundance (maximum annual count during March 1 to June 30 pupping season) of harbor seal pups and counts of suspected or confirmed coyote predation events at MacKerricher State Beach. *Rookery monitoring was limited during the 2020 harbor seal pupping season due to COVID‐19 restrictions, likely resulting in reduced maximum pup and coyote‐killed pup counts.

Across dozens of kill site investigations and harbor seal pup necropsies from 2016 to 2022, we strongly suspected that coyotes were responsible for most or all the predation events due to the presence of canid tracks adjacent to kill sites and drag marks, the presence of coyote scat near seal carcasses, and puncture wounds to the skull and neck consistent with canid predation. To confirm that coyotes were hunting harbor seal pups at the MacKerricher State Beach rookery, we placed one to two motion‐triggered camera traps (Browning Strike Force HD Pro X) in April–May 2023 (16 trap‐nights) and two to four camera traps in April–May 2024 (48 trap‐nights) to document pup predation events. Cameras were mounted to driftwood and programmed to take 20 or 60‐s videos when triggered, with a 30‐s “quiet period” following video capture before another video could be triggered. Throughout 64 trap‐nights (32 nights total), we documented three instances of individual coyotes dragging harbor seal pups into dune vegetation, opening carcasses through the head and neck region, feeding, and removing skulls (Video S1). These events occurred in the evening and early morning hours, between 23:00 and 5:00. Necropsies and kill site investigations conducted the morning following each of these predation events revealed drag marks in the sand, massive hemorrhaging in the neck region, skull punctures, and skull disarticulation from the body consistent with prior events of suspected predation (Figure 1). These lines of corroborating evidence suggest that the harbor seal pups documented on camera were killed by coyotes rather than scavenged, and the consistency of these predation events with over 50 pup carcasses showing similar mortality patterns in 2016–2024 is suggestive that coyotes regularly hunt seal pups in this locality (Figure 2; Appendix S1: Table S1).

We estimated the standard length of harbor seal pups during field necropsies, as direct measurement was often impossible due to skull removal by coyotes. The mean estimated length of coyote‐killed seals was 80.57 ± 0.89 cm (SE; *n* = 54), indicating that most were less than two weeks old and had an estimated body mass of 13–15 kg (Cottrell et al., 2002). For comparison, coyotes in the western United States typically have a body mass between 10 and 14 kg, making each harbor seal pup a substantial food source (Hinton et al., 2019). The seasonality of coyote predation events reflects this trend of coyotes primarily killing young and small harbor seal pups, with 70.9% of predation events occurring within the first 20 days of the pupping season at the MacKerricher rookery (April 14 to May 4; Figure 2A). High rates of coyote predation on small seal pups could indicate that coyotes target this age class or that seal pups become less vulnerable to predation as they age due to increases in body size, vigilance, or escape ability. In addition, coyotes at MacKerricher State Park appear to first remove harbor seal pup skulls and consume brain tissue, potentially because the brain tissue is highly nutritious, easily accessible, and/or can be easily carried to the den for later consumption or for feeding coyote pups. Our cameras documented coyotes returning to seal pup carcasses to scavenge on subsequent evenings, along with other coastal scavengers exploiting the coyote‐killed pinniped carrion including Bald Eagles (*Haliaeetus leucocephalus*), Turkey Vultures (*Cathartes aura*), Common Ravens (*Corvus corax*), gulls (*Larus* spp.), and rodents.

Lastly, to assess whether harbor seal pup predation by coyotes occurs across a larger geographic scale on the California coast, we also gathered observations of coyote–seal interactions from seal rookery monitors, naturalists, and wildlife photographers through word‐of‐mouth networking. From these contributors, we received documentation of four independent direct observations of coyotes successfully hunting harbor seal pups at two rookery sites in Marin County (Drakes Estero and Bolinas Lagoon; Appendix S1: Table S1, Observations 32, 37, 40, and 50). In each of these instances, observers watched one or two coyotes ambushing hauled‐out harbor seals at the edge of exposed intertidal sandbars within coastal lagoons (Figure 3). In all cases, the hunting coyote(s) charged at large aggregations of harbor seal adults and pups, causing seals to flush into the water to escape predation. Coyotes captured seal pups onshore or, in one instance, in shallow water, killing them with bites to the neck.

**FIGURE 3:**
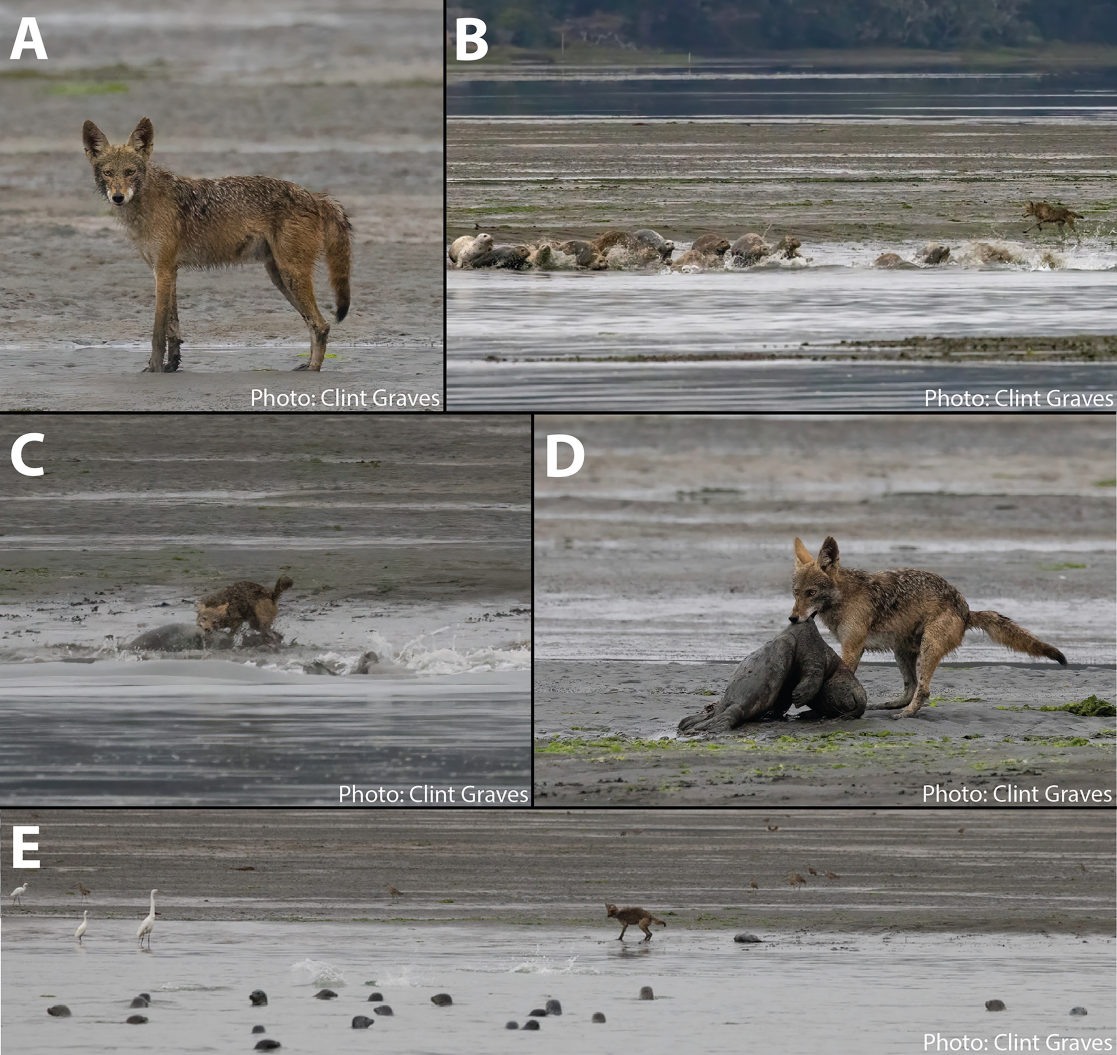
Coyote hunting and killing a harbor seal pup in Bolinas Lagoon on July 4, 2022 (Observation #40). A single coyote (A) was observed ambushing a group of hauled‐out adult harbor seals and pups (B) and killing a harbor seal pup (C, D). Seals flushed into the water to avoid coyote predation (E). Photo credit: Clint Graves.

Coyotes are abundant in California's coastal ecosystems and are known to consume a diverse array of marine resources including seabirds, intertidal invertebrates, fishes, and beach‐cast pinniped carrion (Reid et al., 2018; Rose & Polis, 1998; Zilz et al., 2023). While coyote acquisition of marine resources occurs primarily via scavenging, coyotes have been documented hunting marine mammals in other regions including a sea otter in Alaska (Monnett & Siniff, 1986) and a harp seal in Massachusetts (Way & Horton, 2004). In addition, coyote predation was acknowledged as a source of harbor seal pup mortality in Washington by Steiger et al. (1989). However, aside from the observations and data presented herein, evidence and detailed descriptions of serial pinniped predation by coyotes remain limited.

Pinnipeds are among the largest and most nutritionally valuable parcels of organic matter available to predators in coastal ecosystems (Quaggiotto et al., 2018). Pinniped rookeries, which exhibit predictable aggregations of high‐value prey, can consequently serve as important sites of marine‐to‐terrestrial nutrient subsidies to land‐based pinniped predators (Catenazzi & Donnelly, 2008; Stander, 2019). Along the California coast, pinnipeds can constitute over 20% of the diet of coyotes in areas adjacent to rookeries (Reid et al., 2018). Rookery‐associated marine subsidies may, in turn, drive behavioral and numerical responses in coastal coyote populations (Rose & Polis, 1998). Future efforts to quantify the ecological consequences of pinniped subsidies to coastal coyotes should assess whether pinniped predation modifies coyote space use, behavior, health, abundance, and interspecific interactions.

Coyote predation may also lead to changes in seal behavior and space use in order to reduce predation risk. Harbor seals abandon haul‐outs when they detect terrestrial predators, and the selection of isolated haul‐out sites may serve as a behavioral adaptation to avoid predation by terrestrial consumers (Nordstrom, 2002; Figure 3). Actual or perceived risk of coyote predation may drive harbor seal distributional shifts away from rookeries with predator access, including the mainland sandy beach rookery at MacKerricher State Park. Overall harbor seal abundance has declined at the MacKerricher rookery since 2018, and we have also noticed that the distribution of hauled‐out harbor seals within the rookery has shifted away from the mainland sandy beach onto adjacent rocky intertidal outcroppings that are only connected to the mainland during low tide (Appendix S1: Figure S1). It is possible that between‐ and within‐rookery shifts in harbor seal space use have emerged as a behavioral response to the risk of coyote predation, but these associations between seal redistribution and coyote predation remain anecdotal and do not appear to have limited pup predation.

Harbor seals have used the MacKerricher rookery for decades, so the observed increase in coyote predation on seal pups since 2016 may reflect improved documentation rather than a true rise in predation rates. The rookery monitoring program has focused on counting adult and juvenile seals within the rookery since 2007, while coyotes tend to drag carcasses away from the rookery into nearby vegetation, thereby making predation events less noticeable. Since 2016, enhanced attention to mortality patterns by rookery monitors has likely contributed to better recognition of these events. However, changes in coyote behavior, abundance, alternative prey availability, or other ecological factors could also have led to a genuine increase in predation rates.

Our observations and data indicate that coyotes are an ambush predator of harbor seal pups in multiple regions along the California coast, including one mainland rookery in which coyotes regularly hunt seals during the pupping season. Due to their large body size and predictable availability, harbor seal pups can serve as a substantial food source for coyote predators that may generate cascading ecological effects in terrestrial ecosystems. Coyote predation may also lead to shifts in seal behavior and haul‐out site selection to reduce predation risk. Consequently, coyote predation of harbor seal pups can link the dynamics of marine and terrestrial ecosystems, highlighting the need for future research to uncover the ecological consequences of this previously undescribed predator–prey interaction.

## CONFLICT OF INTEREST STATEMENT

The authors declare no conflicts of interest.

## Supporting information


Appendix S1:

Video S1:

**Video S1** **Metadata:**

## Data Availability

Data and code (Gerraty et al., 2024) are available in Zenodo at https://doi.org/10.5281/zenodo.14502312.
