# Supplementary material for: Coyotes Hunt Harbor Seal Pups on the California Coast
Source: Ecology. 2025 Feb 12;106(2):e70031. doi: 10.1002/ecy.70031 (PMC11815357; doi:10.1002/ecy.70031)
Supplement: Supplementary file 1 — Appendix S1: [file ECY-106-e70031-s001.pdf]

ECOLOGY

Appendix S1

Coyotes hunt harbor seal pups on the California coast

Francis D. Gerraty, Sarah Grimes, Sue Pemberton, Sarah G. Allen, Sarah A. Codde

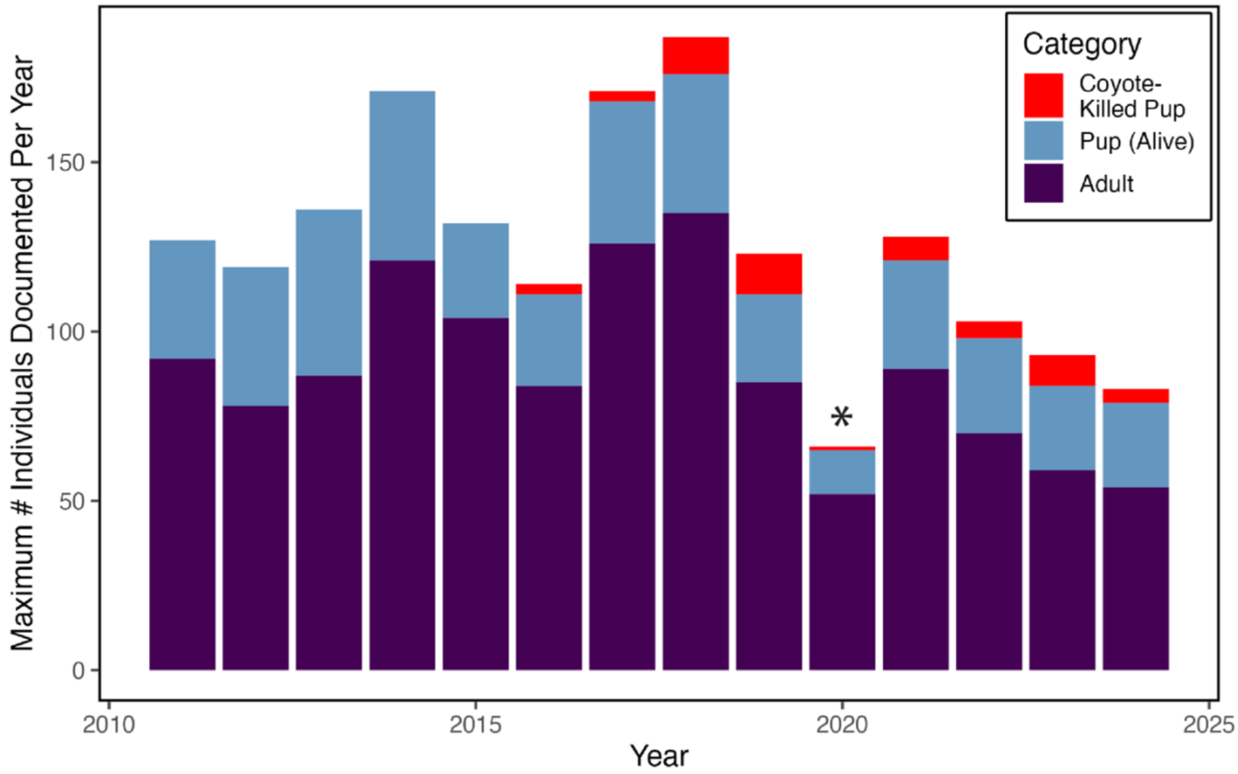

Figure S1: Abundance (maximum annual count during March 1 – June 30 pupping season) of harbor seal adults, pups and counts of suspected or confirmed coyote predation events at MacKerricher State Beach. *Rookery monitoring was limited during the 2020 harbor seal pupping season due to COVID-19 restrictions, likely resulting in reduced maximum adult, pup and coyote-killed pup counts.

Table S1. Confirmed and suspected coyote predation events documented in 2016-2024 at three locations along the California coast: MacKerricher State Park (39.49480 N, -123.79433 W), Drakes Estero (38.03664 N, -122.93826 W), and Bolinas Lagoon (37.92017 N, -122.68110 W).

**Obser-vation #**	**Observation Type**	**Location**	**Date**	**Pup Length (cm)**
1	Suspected coyote predation	MacKerricher State Park	5/24/2016	*80*
2	Suspected coyote predation	MacKerricher State Park	5/24/2016	*80*
3	Suspected coyote predation	MacKerricher State Park	5/24/2016	*80*
4	Suspected coyote predation	MacKerricher State Park	4/24/2017	91
5	Suspected coyote predation	MacKerricher State Park	4/24/2017	83
6	Suspected coyote predation	MacKerricher State Park	4/28/2017	94
7	Suspected coyote predation	MacKerricher State Park	4/14/2018	*75*
8	Suspected coyote predation	MacKerricher State Park	4/15/2018	*77*
9	Suspected coyote predation	MacKerricher State Park	4/15/2018	*79*
10	Suspected coyote predation	MacKerricher State Park	4/16/2018	*80*
11	Suspected coyote predation	MacKerricher State Park	4/16/2018	*77*
12	Suspected coyote predation	MacKerricher State Park	4/27/2018	*82*
13	Suspected coyote predation	MacKerricher State Park	4/30/2018	*79*
14	Suspected coyote predation	MacKerricher State Park	5/1/2018	*75*
15	Suspected coyote predation	MacKerricher State Park	5/3/2018	*82*
16	Suspected coyote predation	MacKerricher State Park	5/17/2018	91
17	Suspected coyote predation	MacKerricher State Park	6/8/2018	*102*
18	Suspected coyote predation	MacKerricher State Park	4/24/2019	83
19	Suspected coyote predation	MacKerricher State Park	4/29/2019	*78*
20	Suspected coyote predation	MacKerricher State Park	4/29/2019	*72*
21	Suspected coyote predation	MacKerricher State Park	5/2/2019	82
22	Suspected coyote predation	MacKerricher State Park	5/2/2019	69
23	Suspected coyote predation	MacKerricher State Park	5/3/2019	*80*
24	Suspected coyote predation	MacKerricher State Park	5/5/2019	67
25	Suspected coyote predation	MacKerricher State Park	5/5/2019	69
26	Suspected coyote predation	MacKerricher State Park	5/6/2019	*79*
27	Suspected coyote predation	MacKerricher State Park	5/10/2019	*79*
28	Suspected coyote predation	MacKerricher State Park	5/13/2019	*80*
29	Suspected coyote predation	MacKerricher State Park	5/24/2019	*78*
30	Suspected coyote predation	MacKerricher State Park	4/30/2020	*80*
31	Suspected coyote predation	MacKerricher State Park	4/22/2021	*77*
32	Confirmed coyote predation	Drakes Estero	4/22/2021	ND
33	Suspected coyote predation	MacKerricher State Park	4/23/2021	81
34	Suspected coyote predation	MacKerricher State Park	4/28/2021	*77*
35	Suspected coyote predation	MacKerricher State Park	4/28/2021	*80*
36	Suspected coyote predation	MacKerricher State Park	5/4/2021	*85*
37	Confirmed coyote predation	Drakes Estero	5/6/2021	ND
38	Suspected coyote predation	MacKerricher State Park	5/13/2021	*75*

39	Suspected coyote predation	MacKerricher State Park	5/14/2021	*83*
40	Confirmed coyote predation	Bolinas Lagoon	4/7/2022	ND
41	Suspected coyote predation	MacKerricher State Park	4/17/2022	*80*
42	Suspected coyote predation	MacKerricher State Park	4/17/2022	*75*
43	Suspected coyote predation	MacKerricher State Park	4/26/2022	*75*
44	Suspected coyote predation	MacKerricher State Park	4/28/2022	*80*
45	Suspected coyote predation	MacKerricher State Park	4/28/2022	*96*
46	Suspected coyote predation	MacKerricher State Park	4/16/2023	*67*
47	Suspected coyote predation	MacKerricher State Park	4/22/2023	*86*
48	Suspected coyote predation	MacKerricher State Park	4/22/2023	*87*
49	Suspected coyote predation	MacKerricher State Park	4/22/2023	*87*
50	Confirmed coyote predation	Drakes Estero	4/22/2023	ND
51	Confirmed coyote predation	MacKerricher State Park	4/24/2023	ND
52	Suspected coyote predation	MacKerricher State Park	5/3/2023	*80*
53	Suspected coyote predation	MacKerricher State Park	5/3/2023	*80*
54	Suspected coyote predation	MacKerricher State Park	5/8/2023	*80*
55	Suspected coyote predation	MacKerricher State Park	5/19/2023	*86*
56	Suspected coyote predation	MacKerricher State Park	4/22/2024	*83*
57	Suspected coyote predation	MacKerricher State Park	4/27/2024	*83*
58	Confirmed coyote predation	MacKerricher State Park	4/30/2024	*84*
59	Confirmed coyote predation	MacKerricher State Park	5/7/2024	*80*

Abbreviations: ND = No data. Notes: Pup length values in italics were estimated due to skull disarticulation from vertebral column.
