# Supplementary material for: Coyotes Hunt Harbor Seal Pups on the California Coast
Source: Ecology. 2025 Feb 12;106(2):e70031. doi: 10.1002/ecy.70031 (PMC11815357; doi:10.1002/ecy.70031)
Supplement: Supplementary file 3 — Video S1 Metadata: [file ECY-106-e70031-s003.pdf]

ECOLOGY

Video S1 Metadata

Coyotes hunt harbor seal pups on the California coast

Francis D. Gerraty, Sarah Grimes, Sue Pemberton, Sarah G. Allen, Sarah A. Codde

Video S1: Camera trap video recordings documenting coyotes dragging harbor seal pups into dune vegetation, opening carcasses through the head and neck region, feeding, and removing skulls. The observation numbers shown in the video match the observation numbers listed in the "Observation #" column of Table S1. Credit for all videos: Francis D. Gerraty.
